# Protection against Omicron BA.1/BA.2 severe disease 0–7 months after BNT162b2 booster

**DOI:** 10.1038/s42003-023-04669-6

**Published:** 2023-03-23

**Authors:** Ofra Amir, Yair Goldberg, Micha Mandel, Yinon M. Bar-On, Omri Bodenheimer, Laurence Freedman, Sharon Alroy-Preis, Nachman Ash, Amit Huppert, Ron Milo

**Affiliations:** 1 Technion - Israel Institute of Technology, Haifa, Israel; 2 The Hebrew University of Jerusalem, Jerusalem, Israel; 3 Department of Plant and Environmental Sciences, Weizmann Institute of Science, Rehovot, Israel; 4 Israel Ministry of Health, Jerusalem, Israel; 5 The Bio-statistical and Bio-mathematical Unit, The Gertner Institute for Epidemiology & Health Policy Research, Sheba Medical Center, Ramat Gan, Israel; 6 The Faculty of Medicine, Tel Aviv University, Tel Aviv, Israel

**Keywords:** Infectious diseases, Vaccines

## Abstract

Following evidence of waning immunity against both infection and severe disease after 2 doses of the BNT162b2 vaccine, Israel began administering a 3rd BNT162b2 dose (booster) in July 2021. Recent studies showed that the 3rd dose provides a much lower protection against infection with the Omicron variant compared to the Delta variant and that this protection wanes quickly. However, there is little evidence regarding the protection of the 3rd dose against Omicron (BA.1/BA.2) severe disease. In this study, we estimate the preservation of immunity from severe disease up to 7 months after receiving the booster dose. We calculate rates of severe SARS-CoV-2 disease between groups of individuals aged 60 and above, comparing those who received two doses at least 4 months previously to those who received the 3rd dose (stratified by the time from vaccination), and to those who received a 4th dose. The analysis shows that protection conferred by the 3rd dose against Omicron severe disease did not wane over a 7-month period. Moreover, a 4th dose further improved protection, with a severe disease rate approximately 3-fold lower than in the 3-dose cohorts.

## Abstract (web summary)

Patient data from the Israeli Ministry of Health demonstrates that a 3rd dose of BNT162b2 is effective in reducing Omicron BA.1/BA.2 severe disease and does not wane over the seven month study period as well as that a fourth dose further improves the protective features of vaccination.

## Introduction

Following a rise in cases due to the Delta variant and evidence of waning immunity against both confirmed infection and severe disease after 2 doses of the BNT162b2 vaccine^1^, Israel began administering a third BNT162b2 dose (booster) in July 2021. The 3rd dose was very effective against both confirmed infections and severe disease caused by the Delta variant^2^. However, recent studies have shown that the 3rd dose provides a much lower protection against infection with the Omicron variant (BA.1/BA.2) and that this protection wanes quickly^3,4^.

While a recently-administered 3rd dose was shown to be effective against severe disease caused by the Omicron variant^5^, it is not yet known how long this protection lasts. A study from the UK estimated 85% vaccine effectiveness against hospitalization for individuals 65 years of age or older 15 weeks after receiving the 3rd dose^6^. A CDC study showed waning of protection against hospitalization from 91% in the first 2 months after the 3rd dose to 78% by the fourth month^7^.

Here, we report on the protection against severe disease with the BA.1/BA.2 Omicron variants in individuals aged 60 or older up to 7 months after receiving a 3rd BNT162b2 dose, during the study period, January 16, 2022, to March 12, 2022. We used Poisson regression to estimate the rate of severe SARS-CoV-2 disease in the following cohorts: individuals who received the 2nd dose at least 4 months before the study ended, individuals who received the 3rd dose (divided into cohorts based on months from vaccination), and individuals who received the 4th dose. The regression model was adjusted for age group (60–69, 70–79, 80+), sex, sector (Arab, General Jewish, Ultra-Orthodox), and epidemiological week.

The follow-up was not extended since severe cases beyond the study period are affected not only by the vaccination but also by extremely widespread and largely undocumented prior infections due to increased use of antigen self-testing, which affected surveillance. In addition, the increased use of preventive medicines (e.g., Paxlovid and Molnupiravir) during this period raises further challenges in estimating vaccine effectiveness against severe disease.

The analysis shows that protection conferred by the 3rd dose against Omicron severe disease did not wane over a 7-month period. Moreover, a 4th dose further improved protection, with a severe disease rate approximately threefold lower than in the 3-dose cohorts.

## Results

Table 1 reports basic descriptive statistics for the main cohorts of interest used in the analysis. The proportion of female days at risk is somewhat higher than that for males, with about 60% in the 2nd dose and 3rd dose up to 4 months cohorts and decreasing to about 55% for the 3rd dose over 5 months and the 4th dose cohorts. There are some differences in the age distribution across the different cohorts, with the proportion of days at risk among those aged 70–80 between 27 and 34% in the 2nd and 3rd dose cohorts and 42% in the 4th dose cohort. Similarly, the proportion of days at risk among those aged 80 and above is between 15 and 22% in the 2nd and 3rd dose cohorts and 26% in the 4th dose cohort. In addition, the distribution of sectors differs between the cohorts, with the proportion of days at risk among the General Jewish population being 63% in the 2nd dose cohort, between 77 and 85% in the 3rd dose cohorts, and 94% in the 4th dose cohort.

**Table 1:** Demographic and clinical characteristics of the different cohorts.

Group		Total	Female	Male	60–69	70–79	80+	General Jewish	Ultra-Orthodox Jewish	Arabs
2 Doses 4+ months *N* = *111,531 Person-days at risk* = 3,858,236	*% person days at risk*	100%	60.80%	39.20%	53.50%	28.00%	18.50%	63.20%	5.70%	31.10%
	*# severe Covid-19 (rate)*	469 (12.2)	236 (10.1)	233 (15.4)	100 (4.8)	142 (13.1)	227 (31.9)	281 (11.5)	18 (8.2)	170 (14.2)
3 Doses 0–1 months *N* = *31,955 Person-days at risk* = 446,008	*% person days at risk*	100%	59.80%	40.20%	47.20%	30.00%	22.80%	77.80%	5.10%	17.10%
	*# severe Covid-19 (rate)*	29 (6.5)	12 (4.5)	17 (9.5)	5 (2.4)	7 (5.2)	17 (16.7)	22 (6.3)	2 (8.8)	5 (6.5)
3 Doses 1–2 months *N* = *40,172 Person-days at risk* = 524,213	*% person days at risk*	100%	60.40%	39.60%	51.00%	29.70%	19.30%	77.70%	4.90%	17.40%
	*# severe Covid-19 (rate)*	18 (3.4)	11 (3.5)	7 (3.4)	3 (1.1)	5 (3.2)	10 (9.9)	12 (2.9)	1 (3.9)	5 (5.5)
3 Doses 2–3 months *N* = *43,689 Person-days at risk* = 683,876	*% person days at risk*	100%	61.70%	38.30%	51.70%	29.10%	19.20%	76.90%	4.60%	18.50%
	*# severe Covid-19 (rate)*	30 (4.4)	16 (3.8)	14 (5.3)	4 (1.1)	8 (4)	18 (13.7)	25 (4.8)	1 (3.2)	4 (3.2)
3 Doses 3–4 months *N* = *96,809 Person-days at risk* = 2,286,002	*% person days at risk*	100%	59.60%	40.40%	57.00%	27.40%	15.60%	78.10%	4.20%	17.70%
	*# severe Covid-19 (rate)*	104 (4.5)	45 (3.3)	59 (6.4)	17 (1.3)	29 (4.6)	58 (16.3)	76 (4.3)	4 (4.2)	24 (5.9)
3 Doses 4–5 months *N* = *253,259 Person-days at risk* = 7,262,548	*% person days at risk*	100%	57.00%	43.00%	56.40%	28.80%	14.80%	81.60%	4.40%	13.90%
	*# severe Covid-19 (rate)*	353 (4.9)	155 (3.7)	198 (6.3)	62 (1.5)	111 (5.3)	180 (16.7)	270 (4.6)	19 (5.9)	64 (6.3)
3 Doses 5–6 months *N* = *683,273 Person-days at risk* = 13,018,739	*% person days at risk*	100%	54.70%	45.30%	51.80%	32.40%	15.80%	84.60%	4.80%	10.60%
	*# severe Covid-19 (rate)*	561 (4.3)	220 (3.1)	341 (5.8)	89 (1.3)	171 (4.1)	301 (14.7)	458 (4.2)	28 (4.5)	75 (5.4)
3 Doses 6–7 months *N* = *521,400 Person-days at risk* = 5,942,383	*% person days at risk*	100%	53.60%	46.40%	49.40%	34.40%	16.20%	85.60%	5.00%	9.50%
	*# severe Covid-19 (rate)*	75 (1.3)	21 (0.7)	54 (2)	13 (0.4)	24 (1.2)	38 (3.9)	49 (1)	4 (1.4)	22 (3.9)
4 Doses 0–2 months *N* = *515,029 Person-days at risk* = 20,924,765	*% person days at risk*	100%	52.60%	47.40%	32.00%	41.70%	26.30%	93.90%	2.70%	3.50%
	*# severe Covid-19 (rate)*	280 (1.3)	113 (1)	167 (1.7)	28 (0.4)	83 (1)	169 (3.1)	252 (1.3)	6 (1.1)	22 (3)

Risk days and infections are calculated for the study period, January 16–March 12, 2022. Severe disease cases were defined as a severe illness occurring within 14 days of an infection confirmed during the study period. Rates are per 100,000 person-days. The total number (N) of individuals that contributed at least one risk day to a cohort appears in the first column where an individual may contribute to more than one cohort. The table presents in detail the proportion of person-days at risk (for the confirmed infection analysis) instead of the number of individuals, as people move between cohorts. The table presents the percentage of days at risk contributed for each sub-population (e.g., female and male) in each cohort.

**Fig. 1:**
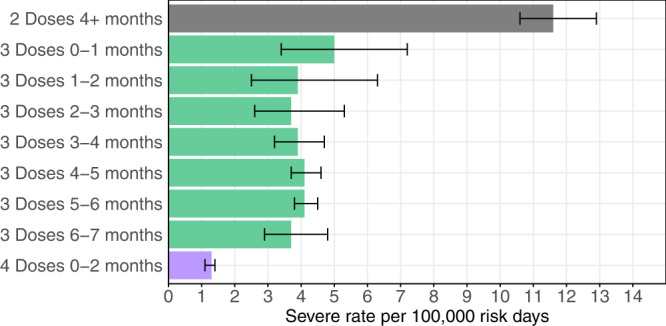
Adjusted rates of severe illness. Adjusted rates of severe illness per 100,000 risk days obtained from Poisson regression analysis for the study period January 16, 2022–March 12, 2022, adjusted for age category (60–69, 70–79, 80+), sex, sector, and exposure (based on epidemiological week). Error bars represent 95% confidence intervals, which are not adjusted for multiplicity.

The rates of severe disease, and their ratios, estimated from the Poisson regression are summarized in Table 2 and Fig. 1. The protection conferred by the 3rd dose against Omicron severe disease did not show signs of waning over the first 7 months since vaccination, with rates of severe disease at ~4 per 100,000 risk days in all 3rd dose cohorts. This rate was approximately threefold lower than that in the 2nd dose cohort. The 4th dose further increased protection, with a severe disease rate approximately threefold lower than that in the 3rd dose cohorts.

**Table 2:** Results of the Poisson regression analysis for severe disease.

Group (days from vaccination)	Severe cases (person-days at risk)	Adjusted Rate of severe disease per 100,000 person-days at risk [95% CI]	Expected number of severe cases per month [95% CI]	Inverse of Adjusted Rate Ratio relative to 2nd dose cohort [95% CI]
2nd dose (4+ months)	469 (3,858,236)	11.6 [10.6, 12.9]	4100 [3746, 4559]	Ref
3rd dose (0–1 months)^a^	29 (446,008)	5.0 [3.5, 7.2]	1767 [1237, 2545]	2.3 [1.6, 3.4]
3rd dose (1–2 months)	18 (524,213)	3.9 [2.5, 6.3]	1378 [884, 2227]	2.9 [1.8, 4.7]
3rd dose (2–3 months)	30 (683,876)	3.7 [2.6, 5.3]	1308 [919, 1873]	3.1 [2.2, 4.6]
3rd dose (3–4 months)	104 (2,286,002)	3.9 [3.2, 4.7]	1378 [1131, 1661]	3.0 [2.4, 3.7]
3rd dose (4–5 months)	353 (7,262,548)	4.1 [3.7, 4.6]	1449 [1308, 1626]	2.8 [2.5, 3.3]
3rd dose (5–6 months)	561 (13,018,739)	4.1 [3.8, 4.5]	1449 [1343, 1590]	2.8 [2.5, 3.2]
3rd dose (6–7 months)	75 (5,942,383)	3.7 [2.9, 4.8]	1308 [1025, 1696]	3.1 [2.4, 4.1]
4th dose(0–2 months)^a^	280 (20,924,765)	1.3 [1.1, 1.4]	459 [389, 495]	9.2 [7.9, 10.7]

The first column presents the number of severe cases during the study period with person-days at risk in parentheses. The second column shows the adjusted rate of severe disease per 100,000 person-days. The third column presents the expected number of severe cases per month, assuming all the study population belonged to the corresponding cohort. The last column presents the adjusted rate ratios compared to the 2nd dose cohort.

^a^The first 13 days are excluded.

Two sensitivity analyses, one allowing 21 (rather than the standard 14) days from a positive COVID test for severe disease to develop, and one stratified by age group, yielded rate estimates similar to those obtained in the main analysis.

## Discussion

Studies have shown that the protection of a 2-dose BNT162b2 vaccine against both confirmed infection and severe disease wanes over time^1^. A recently-administered 3rd dose was shown to not just restore the initial level of protection of the 2-dose vaccine but exceed it^8^. Nevertheless, the level of protection of a 3rd BNT162b2 dose against confirmed Omicron BA.1/BA.2 infections was found to be substantially lower than against the Delta variant and to wane quickly. Our analysis shows that the effectiveness of the 3rd dose in preventing severe disease substantially exceeds that of the 2nd dose. Moreover, unlike the protection of the 2-dose vaccine, this protection does not appear to wane over the first 7 months since vaccination. Furthermore, our analysis regarding the protection against severe disease conferred by a 4th dose compared to the 3rd dose which showed an approximately threefold lower rate of severe disease in the 4th dose cohort aligns with a previous study^9^ that reported a 3.5-fold lower rate.

The results of the current study are aligned with studies that assess the immune levels and their durability following a 3rd BNT162b2 dose. Specifically, a large study from Israel following the serum of health careworks found that after the 3rd dose the humoral response declined slower than after the 2nd dose, started from a higher level in both IgG and neutralizing antibodies^10^. More so, the study also found that the avidity of the antibodies after the 3rd dose is higher than after the second dose^10^. In another study from Singapore showed that the total S-Ab/IgG/N-Ab levels increased post-booster compared to a 2nd dose, with IgG/N-Ab having a longer half-life^11^. Evidence from Japan also suggests that the 3rd dose conferred higher durable antibody titers^12^.

To date, this study reports the longest follow-up following administration of a 3rd dose, as Israel was the first country to administer a 3rd dose. Our analysis was based on a nationwide dataset that included data on more than 1 million elderly individuals. Severe cases were determined in a consistent way according to the NIH definition (see method) and were identified from daily reports issued by all hospitals in Israel. As such, the data were complete and representative of the entire elderly population in Israel.

The results of this study differ from the analysis of a recent CDC study^7^. The CDC study, which analyzed hospitalizations rather than severe cases, showed substantial waning 4 months after receiving the 3rd dose. However, there were only 765 individuals hospitalized over 4 months after receiving the 3rd dose. In addition, hospitalization is a measure that is not as specific as severe disease, hence can include a wide range of severity levels^13^.

This observational study has several limitations. First, there may be differences in risk of severe disease between the different cohorts. This limitation is addressed by adjusting for confounders such as age, sex, sector, and exposure. However, some risk differences could not be adjusted for. For instance, individuals who received the 3rd dose early may have been more likely to suffer from coexisting conditions. This could potentially bias the results toward under-estimation of vaccine effectiveness. Information on coexisting conditions is not recorded in the national database, and could not be adjusted for. Differences in coexisting conditions could also be associated with differential treatment with antiviral drugs such as Paxlovid and Molnupiravir, which could have affected the results^14^. In addition, individuals may have undocumented prior infections leading to hybrid immunity. As such hybrid immunity increases protection^15^, undocumented infections can severely bias the results. Moreover, the rate of prior infection is expected to be higher for people who received fewer vaccine doses as they are less protected, further biasing the results^16^.

Similar limitations arise in the comparison of the 3rd dose cohorts to the 4th dose cohort^17^. Specifically, individuals who tend to be more careful might also be more likely to take the 4th dose (leading to overestimation of the effect of the 4th dose). On the other hand, people who are at higher risk (e.g., due to comorbidities) might have a stronger tendency to both take the 4th dose and experience severe outcomes (leading to under-estimation of the effect of the 4th dose). Nevertheless, our results align with a previous study on the protection conferred by the 4th dose in Israel^9^ that overcomes the aforementioned behavioral biases. This latter study used an internal control group, comprised of individuals during days 3–7 after receiving the 4th dose, that is, before the booster dose was expected to have an effect, and obtained estimates for the protection against severe disease similar to ours.

During the study period, the dominant variant was Omicron sublineages BA.1/BA.2. Estimating the protection against Omicron BA.4/BA.5 severe disease and potential waning of the 3rd dose beyond 7 months is especially difficult due to missing infection data. Many, possibly most, individuals from all relevant cohorts (2nd, 3rd, and 4th dose) have likely been infected during the unprecedented BA.1/BA.2 Omicron wave. These infections are often undocumented as during this wave people increasingly self-tested at home using antigen tests (which have remained largely undocumented). In addition, during the BA.4/BA.5 wave, Israel greatly expanded the administration of Paxlovid and Molnupiravir to persons testing positive, reducing severe cases regardless of vaccination status. Thus, when other factors (e.g., ubiquitous hybrid immunity, preventive medication) reduce the risk of severe disease, the level of protection provided by vaccination and its waning become extremely difficult to estimate from population data. These challenges with respect to the estimation of protection during the BA.4/BA.5-dominant periods are shared globally. Hence, it is hard to obtain any reliable estimation of 3rd dose waning beyond the follow-up of 7 months reported in this paper.

The findings of the current study can support policy-making. The study suggests an extended duration of protection of the 3rd BNT162b2 dose against severe disease and the added protection of a 4th dose to risk groups. While it is still unclear what are the long-term effects of the 4th dose and the protection conferred by the new bivalent vaccines^18^, our findings can inform decisions regarding the timing of administration of additional booster doses depending on individual risk factors.

## Methods

### Description of the data

The Ministry of Health (MOH) in Israel collects all COVID-19-related variables in a central database. These include data on all institutional PCR and antigen tests (which are given free of charge) and results, vaccination dates and type of vaccine (almost all received the Pfizer-BioNTech vaccine), daily clinical status of all COVID-19-hospitalized patients, and COVID-19-related deaths. Specifically, the data used for conducting this study included vaccination dates, PCR and state-regulated rapid antigen tests (dates and results), hospital admission dates (if relevant), clinical severity status (severe disease or death), and demographic variables such as age, sex, and demographic group (General Jewish, Arab, ultra-Orthodox Jewish).

The data for the study were retrieved on March 26, 2022, with the outcome being severe illness due to Covid-19 during 14 days following a confirmed infection (defined using the NIH definition^19^ as a resting respiratory rate of more than 30 breaths per minute, oxygen saturation of <94% while breathing ambient air, or a ratio of partial pressure of arterial oxygen to fraction of inspired oxygen of <300). Thus, the study period was set from January 16, 2022, to March 12, 2022, to allow for 14 days of follow-up from infection to severe disease. Those who died from COVID-19 during the first 14 days after confirmed infection were also counted as severe disease cases in our analysis. Surveillance of COVID-19-associated hospitalizations is continuously performed by the MOH. Data from all hospitals are updated daily, and often twice a day. In accordance with national guidelines, healthcare providers report all hospitalizations and deaths among individuals with laboratory-confirmed SARS-CoV-2 infection. Quality assurance of data was performed extensively over the course of the pandemic. The data are monitored daily by the MOH, and are continuously used for public health decision-making.

### Study design and population

**Fig. 2:**
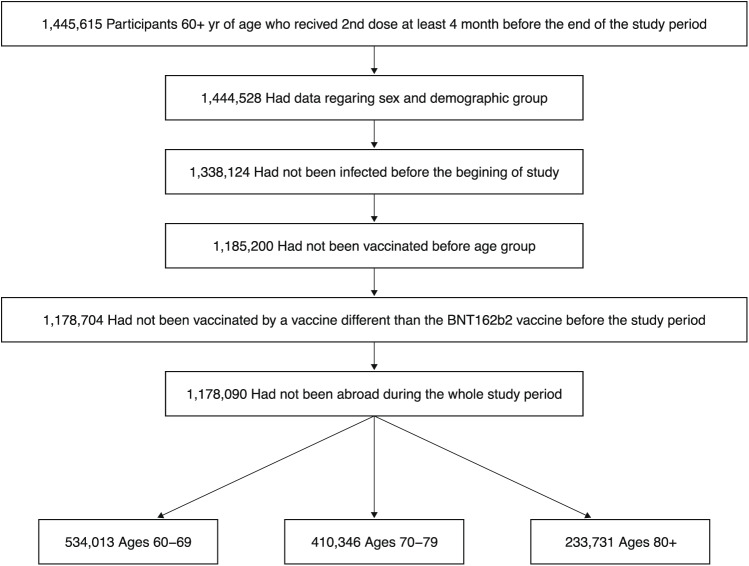
Study population. The participants in the study included persons who were 60 years of age or older, who received 2nd dose at least 4 months before the end of the study, who were not infected by SARS-CoV-2 before the study period, had available data regarding sex and demographic sector, had not stayed abroad during the whole study period, and had not been vaccinated with a vaccine different from BNT162b2 before the study period.

We conducted an observational study during Israel’s fifth wave, which was Omicron-dominant^20^, with both BA.1 and BA.2 sublineages present (initially BA.1 dominated, later BA.2 dominated). The study included persons who were 60 years of age or older, received their 2nd dose at least 4 months before the end of the study, did not have a documented infection by SARS-CoV-2 before the study period, had available data regarding sex and demographic sector, had not stayed abroad during the whole study period, and had not been vaccinated with a vaccine different from BNT162b2 before the study period (see Fig. 2).

### Statistics and reproducibility

**Fig. 3:**
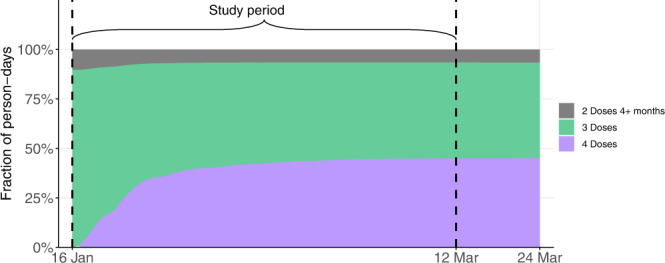
Vaccination dynamics. Vaccination dynamics of people aged 60 or above during the study period. The dashed vertical lines represent the study period, between January 16, 2022, and March 12, 2022.

We estimated the adjusted rates of severe disease in nine cohorts: people who received only 2 doses of the vaccine and for whom at least 4 months had passed since receiving the second dose, people who received a 3rd vaccine dose, divided into 7 cohorts based on the number of months that passed since the administration of that dose, and people who recently received a 4th vaccine dose. Figure 3 shows the vaccination dynamics during the study period, and Table 2 reports basic descriptive statistics for the different cohorts. Individuals moved from one cohort to another after receiving a new vaccine or according to the time elapsed from the 3rd dose. Days at risk and severe disease cases occurring between vaccination (3rd or 4th dose) and 13 days after it were excluded, as they could not yet be associated with the new dose. Risk days for individuals who traveled abroad were not counted while they were outside of Israel.

A Poisson regression model was used to estimate the adjusted rate of severe disease per 100,000 risk days. The model included covariates adjusting for age group (60–69, 70–79, 80+), sex, sector (Arab, General Jewish, Ultra-Orthodox), and epidemiological week. We further conducted two sensitivity analyses. First, we included severe disease occurring up to 21 days following a confirmed infection. Second, we estimated the adjusted rates of severe disease within each age group (60–69, 70–79, and 80+).

### Reporting summary

Further information on research design is available in the Nature Portfolio Reporting Summary linked to this article.

## Supplementary information

Description of Additional Supplementary Files

Supplementary Code 1

Supplementary Data 1

Reporting Summary

## Data Availability

The data that support the findings of this study are available from the Israeli Ministry of Health but restrictions apply to the availability of these data and are not publicly available. Data are however available from the authors upon reasonable request and with permission of the Israeli Ministry of Health. The numeric data for creating the figures in the paper are included in Supplementary Data 1.

## Code availability

The code for creating the figures in the paper is included in Supplementary Code 1.
