# Supplementary material for: Protection against Omicron BA.1/BA.2 severe disease 0–7 months after BNT162b2 booster
Source: Commun Biol. 2023 Mar 23;6:315. doi: 10.1038/s42003-023-04669-6 (PMC10035472; doi:10.1038/s42003-023-04669-6)
Supplement: Supplementary file 1 — Description of Additional Supplementary Files [file 42003_2023_4669_MOESM1_ESM.pdf]

Description of Additional Supplementary Files

File Name: Supplementary data 1

Description: The numeric data for creating the figures in the paper.

File Name: Supplementary Code 1

Description: The code for creating the figures in the paper.
