# Supplementary material for: Protection against Omicron BA.1/BA.2 severe disease 0–7 months after BNT162b2 booster
Source: Commun Biol. 2023 Mar 23;6:315. doi: 10.1038/s42003-023-04669-6 (PMC10035472; doi:10.1038/s42003-023-04669-6)
Supplement: Supplementary file 4 — Reporting Summary [file 42003_2023_4669_MOESM4_ESM.pdf]

nature portfolio

Corresponding author(s): Yair Goldberg

Last updated by author(s): Dec 7, 2022

Reporting Summary

Nature Portfolio wishes to improve the reproducibility of the work that we publish. This form provides structure for consistency and transparency in reporting. For further information on Nature Portfolio policies, see our Editorial Policies and the Editorial Policy Checklist.

Statistics

For all statistical analyses, confirm that the following items are present in the figure legend, table legend, main text, or Methods section.

n/a	Confirmed	
☐	☒	The exact sample size (*n*) for each experimental group/condition, given as a discrete number and unit of measurement
☒	☐	A statement on whether measurements were taken from distinct samples or whether the same sample was measured repeatedly
☒	☐	The statistical test(s) used AND whether they are one- or two-sided<br>*Only common tests should be described solely by name; describe more complex techniques in the Methods section.*
☐	☒	A description of all covariates tested
☐	☒	A description of any assumptions or corrections, such as tests of normality and adjustment for multiple comparisons
☐	☒	A full description of the statistical parameters including central tendency (e.g. means) or other basic estimates (e.g. regression coefficient) AND variation (e.g. standard deviation) or associated estimates of uncertainty (e.g. confidence intervals)
☒	☐	For null hypothesis testing, the test statistic (e.g. *F*, *t*, *r*) with confidence intervals, effect sizes, degrees of freedom and *P* value noted<br>*Give P values as exact values whenever suitable.*
☒	☐	For Bayesian analysis, information on the choice of priors and Markov chain Monte Carlo settings
☒	☐	For hierarchical and complex designs, identification of the appropriate level for tests and full reporting of outcomes
☒	☐	Estimates of effect sizes (e.g. Cohen's *d*, Pearson's *r*), indicating how they were calculated

Our web collection on statistics for biologists contains articles on many of the points above.

Software and code

Policy information about availability of computer code

Data collection	The data for this study was provided by the Israeli Ministry of Health
Data analysis	Data analysis was done in R, using standard data analysis packages (dplyr) and regression analysis

For manuscripts utilizing custom algorithms or software that are central to the research but not yet described in published literature, software must be made available to editors and reviewers. We strongly encourage code deposition in a community repository (e.g. GitHub). See the Nature Portfolio guidelines for submitting code & software for further information.

Data

Policy information about availability of data

All manuscripts must include a data availability statement. This statement should provide the following information, where applicable:

- Accession codes, unique identifiers, or web links for publicly available datasets
- A description of any restrictions on data availability
- For clinical datasets or third party data, please ensure that the statement adheres to our policy

The data that support the findings of this study are available from the Israeli Ministry of Health but restrictions apply to the availability of these data and are not publicly available. Data are however available from the authors upon reasonable request and with permission of the Israeli Ministry of Health.

Human research participants

Policy information about studies involving human research participants and Sex and Gender in Research.

Reporting on sex and gender	We report sex as it appears in the Ministry of Health dataset.
Population characteristics	The data contained basic demographic data and information on dates of vaccinations, if received, and dates and results of all institutional PCR or Antigen tests performed from March 1, 2020, and on. For individuals with a positive test, the table contained information on the maximum severity status throughout the course of the disease (hospitalization, severe disease, death).
Recruitment	NA
Ethics oversight	The study was approved by the Institutional Review Board of the Sheba Medical Center. Helsinki approval number: SMC-8228-21

Note that full information on the approval of the study protocol must also be provided in the manuscript.

Field-specific reporting

Please select the one below that is the best fit for your research. If you are not sure, read the appropriate sections before making your selection.

☒ Life sciences ☐ Behavioural & social sciences ☐ Ecological, evolutionary & environmental sciences

For a reference copy of the document with all sections, see nature.com/documents/nr-reporting-summary-flat.pdf

Life sciences study design

All studies must disclose on these points even when the disclosure is negative.

Sample size	This is an observational cohort study based on data on all population in Israel in the studied age groups.
Data exclusions	The study included persons who were 60 years of age or older, who received their 2nd dose at least four months before the end of the study, who did not have a documented infection by SARS-CoV-2 before the study period, had available data regarding sex and demographic sector, had not stayed abroad during the whole study period, and had not been vaccinated with a vaccine different from BNT162b2 before the study period
Replication	NA - this is an observational study based on data on all population in Israel in the studied age groups.)
Randomization	NA
Blinding	NA

Reporting for specific materials, systems and methods

We require information from authors about some types of materials, experimental systems and methods used in many studies. Here, indicate whether each material, system or method listed is relevant to your study. If you are not sure if a list item applies to your research, read the appropriate section before selecting a response.

Materials & experimental systems

n/a	Involved in the study
☒	☐ Antibodies
☒	☐ Eukaryotic cell lines
☒	☐ Palaeontology and archaeology
☒	☐ Animals and other organisms
☒	☐ Clinical data
☒	☐ Dual use research of concern

Methods

n/a	Involved in the study
☒	☐ ChIP-seq
☒	☐ Flow cytometry
☒	☐ MRI-based neuroimaging
